# GATA2 up-regulation restores androgen receptor chromatin association and advances darolutamide resistance in prostate cancer

**DOI:** 10.1016/j.gendis.2024.101508

**Published:** 2024-12-28

**Authors:** Tianyi Zhou, Chengtai Yu, Yali Han, Bin He, Qin Feng

**Affiliations:** a Center for Nuclear Receptor and Cell Signaling, Department of Biology and Biochemistry, University of Houston, Houston, TX 77204, USA; b Immunobiology & Transplant Science Center, Department of Surgery and Urology, Houston Methodist Research Institute, Houston, TX 77030, USA

Prostate cancer is one of the most prevalent cancers in men, and there is no cure when it advances to a late stage. Antiandrogens are routinely used in clinics for prostate cancer treatment, and darolutamide (Daro) is the latest FDA-approved antiandrogen drug.^1^ Despite its efficacy, potential drug resistance poses significant challenges in the clinical setting. This study seeks to uncover the molecular mechanisms behind darolutamide resistance and identify potential therapeutic targets to overcome this resistance.

We developed a DaroR (darolutamide-resistant) LNCaP cell model by progressively raising Daro doses to 10 μM and culturing them for 12 months to ensure resistance. Morphologically, DaroR cells exhibited a flattened shape with neurite-like extensions, distinguishing them from the original LNCaP cells (Fig. S1A). Given Daro's potency as an androgen receptor (AR) antagonist, we examined whether the resistant cells maintained their reliance on AR's transcriptional activity. We compared four sample groups: DaroR cells, parental LNCaP cells treated with DMSO (vehicle), 10 nM R1881 (R1881), or 10 μM Daro for a duration of 24 h. Western blot analysis revealed a slight increase in AR protein levels in the resistant cells (Fig. S1B). We then performed chromatin immunoprecipitation sequencing (ChIP-seq) of AR to examine its cistrome in these samples and used Venn diagrams to show their AR binding peaks (Fig. 1A). Transient treatment of Daro significantly reduced AR chromatin binding to 263 peaks, compared with 8128 peaks in R1881-treated cells. However, DaroR cells showed a marked increase in AR chromatin binding, with 36.9% of these sites (2120 out of 5750) matching those in R1881-treated cells, indicating a possible adaptation in DaroR cells allowing AR to regain chromatin binding.

**Figure 1:**
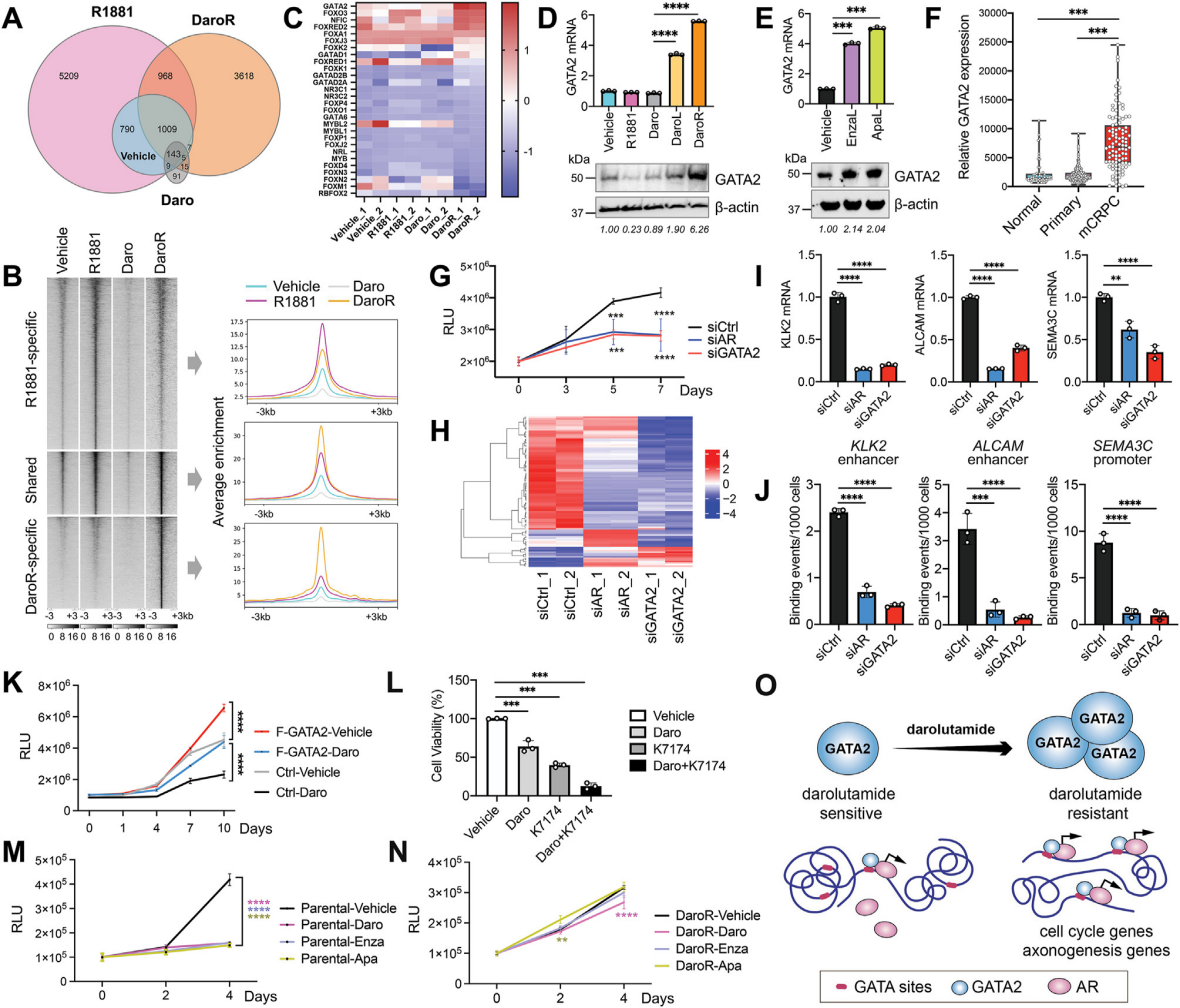
GATA2 up-regulation restores androgen receptor chromatin association and advances darolutamide resistance in prostate cancer. **(A)** Venn diagram shows the overlap between androgen receptor (AR) binding peaks originating from the chromatin immunoprecipitation sequencing (ChIP-seq) of the four specified cell samples, including the parental LNCaP cells treated with DMSO (vehicle), 1 nM R1881 (R1881), or 10 μM darolutamide (Daro) for 24 h, and DaroR LNCaP cells. **(B)** Heatmap and mean signal plot of AR ChIP-seq read coverage at the locations of R1881-specific, DaroR-specific, and jointly occupied/shared sites. The heat maps have been sorted in descending order based on the AR ChIP-seq signal. The average AR binding signal near transcription start sites across these four samples is shown on the right. **(C)** GATA2 expression is elevated in DaroR cells. The heatmap illustrating the expression of transcription factors identified from PscanChIP analysis identifies overrepresented transcription factors in close proximity to the AR binding sites. **(D)** Quantification of mRNA and protein levels of GATA2 in LNCaP cells. DaroL, 3 days of treatment with 10 μM Daro. **(E)** Quantification of mRNA and protein levels of GATA2 in LNCaP cells treated with enzalutamide and apalutamide. EnzaL, 3 days of treatment with 10 μM enzalutamide; ApaL, 3 days of treatment with 10 μM apalutamide. **(F)** GATA2 gene expression in the normal, primary tumor, and metastatic castration-resistant prostate cancer (mCRPC) tissues in TCGA_PRAD and WCDT_MCRPC datasets. Normal, *n* = 52. Tumor, *n* = 500. mCRPC, *n* = 99. **(G)** GATA2 or AR knockdown in DaroR cells by siRNA. The DaroR cells were cultured in a medium containing 10 μM of Daro. Cell growth assay performed after siRNA treatment. The *X*-axis denotes days after siRNA transfection. *n* = 3; error bars, standard deviation. **(H)** The clustering of the differential gene expression heatmap highlighted the overlap in differentially expressed genes between cells treated with siGATA2 and siAR. **(I)** The mRNA levels of AR-regulated genes upon siGATA2 and siAR knockdown in DaroR cells. *n* = 3; error bars, standard deviation. **(J)** Determination of AR chromatin association in designated genomic regions via ChIP-qPCR in DaroR cells treated with siGATA2 or siAR. *n* = 3; error bars, standard deviation. **(K)** Generation of LNCaP cells stably expressing Flag-tagged GATA2. The control (Ctrl) cells were created using the lentiviral backbone vector. GATA2-overexpressing cells and their paired control cells were treated with either DMSO (vehicle) or 10 μM Daro, and cell growth was determined on the indicated days. *n* = 3; error bars, standard deviation. **(L)** Assessment of cell viability in LNCaP cells 3 days after treatment with 10 μM Daro and the GATA2 inhibitor K7174 (10 μM). **(M)** Cell growth in LNCaP parental cells after treatment with 10 μM antiandrogens, including Daro, enzalutamide (Enza), and apalutamide (Apa). The vehicle used was DMSO. **(N)** DaroR cells were treated the same as in (M), and cell growth was determined. **(O)** GATA2 is a key molecule to Daro resistance. Treatment with antiandrogen results in an elevated expression of GATA2. In Daro-sensitive cells, the chromatin association of AR might be constrained due to the limited availability of GATA2. In contrast, in DaroR cells, the up-regulation of GATA2 facilitates the reassociation of AR to chromatin. This is likely achieved by leveraging the pioneer factor activity of GATA2 to make specific chromatin areas more accessible, leading to the expression of cell cycle-related genes and axonogenesis genes.

We further compared AR ChIP-seq read coverage, categorizing binding sites into three groups: unique to R1881-treated cells (*n* = 5209), exclusive to DaroR cells (*n* = 3618), and shared by both (*n* = 2120) (Fig. 1B). Interestingly, AR binding was considerably stronger at DaroR-specific sites, indicating enhanced AR binding at these sites emerged during resistance development. Figure S1C presents a selection of representative genomic loci that harbor AR-binding peaks with R1881-specific, DaroR-specific, or shared sites. Notably, in DaroR cells, AR binding reappeared at KLK2 but not at FKBP5, a result confirmed by AR ChIP-qPCR (Fig. S1D). Accordingly, while R1881 enhanced both FKBP5 and KLK2 expression and Daro treatment reduced them, only KLK2 expression was restored in DaroR cells (Fig. S1E), indicating AR binding restoration at certain genes triggers their re-expression. In contrast, for DaroR-specific genes, such as *ALCAM*, a *de novo* AR binding site is identified upstream of the *ALCAM* gene (Fig. S1C). Our findings show an elevation in *ALCAM* gene expression in DaroR cells (Fig. S1E), which supports the idea that AR recovers its transcriptional activity and promotes advanced tumor development upon the establishment of resistance. This is significant since elevated ALCAM expression is linked to a poorer prognosis and plays a crucial role in the growth and bone metastasis of prostate cancer.^2^^,^^3^

Next, we conducted an RNA-sequencing analysis of these samples and identified 2778 up-regulated and 3087 down-regulated genes differentially expressed in DaroR cells (Fig. S2A). Additionally, we identified 2579 androgen-responsive genes, with 1433 activated and 1146 repressed in R1881-treated versus control cells (Fig. S2B). Within them, 57.1 % of androgen-induced and 80.4 % of androgen-repressed genes were respectively activated or repressed in DaroR cells, suggesting a substantial reactivation of androgen-responsive genes. Gene Set Enrichment Analysis (GSEA) further confirmed a significant correlation with the androgen response pathway, demonstrating a robust recovery of androgen-responsive gene expression in DaroR cells (Fig. S2C).

Consistent with the change in cell morphology, Gene Ontology (GO) enrichment analysis on DaroR cells revealed an up-regulation in axonogenesis pathways (Fig. S2D, E). Notably, class 3 semaphorins (SEMA3s), crucial for axonal guidance, emerged as top genes. SEMA3A is linked to enhancing cancer cell therapy resistance,^4^ and SEMA3C is believed to activate receptor tyrosine kinases and induce AR via a ligand-independent pathway.^5^ AR ChIP-seq analysis showed unique binding peaks for SEMA3C in DaroR cells, suggesting specific AR interaction with SEMA3C's enhancer region (Fig. S2F). This was further supported by ChIP-qPCR and elevated SEMA3C mRNA levels in DaroR cells (Fig. S2G, H), indicating the critical role of SEMA3 pathways in DaroR cell gene expression.

To understand how AR restores chromatin binding in DaroR cells, we used PscanChIP analysis and identified GATA2 as the prominent transcription factor near the AR sites with the highest expression level in DaroR cells (Fig. 1C). GATA2 is a pioneer factor for AR's chromatin access, and its elevated expression suggests it may contribute to chromatin structure opening and AR reassociation in DaroR cells.

We validated that GATA2 mRNA and protein levels were significantly elevated in LNCaP cells after long-term Daro treatment, with a pronounced increase in DaroR cells (Fig. 1D). This up-regulation was consistent across other AR-positive prostate cancer cell lines and induced by second-generation antiandrogens enzalutamide and apalutamide, indicating a general effect of antiandrogen treatment on GATA2 expression (Fig. 1E; Fig. S3). Additionally, GATA2 levels were elevated in enzalutamide-resistant LNCaP cells but not in cells expressing AR-V7 (Fig. S4A). Clinical data further showed GATA2 overexpression in metastatic castration-resistant prostate cancer tumors compared with normal and primary tumors (Fig. 1F), similarly in advanced and castration-resistant prostate cancer samples from another study (Fig. S4B). Therefore, GATA2 up-regulation is likely a consistent response to extended antiandrogen treatment across various models.

GATA2 up-regulation and AR chromatin-binding restoration suggest their joint role in the proliferation of DaroR cells under antiandrogen treatment. DaroR cells with siGATA2 or siAR knockdown grew significantly slower than control knockdown DaroR cells, providing direct evidence that reduced GATA2 or AR expression in DaroR cells restores Daro sensitivity (Fig. 1G; Fig. S5A, B). RNA sequencing showed that GATA2 and AR regulate similar molecular pathways, particularly the cell cycle pathway, indicating their overlapping role in resistance development (Fig. 1H; Fig. S5C, D). Knockdown of GATA2 or AR also decreased the expression of AR-targeted genes in DaroR cells, such as *KLK2*, *ALCAM*, and *SEMA3C*, confirming their cooperative action (Fig. 1I). ChIP-PCR experiments further demonstrated that GATA2 is crucial for AR's chromatin association, as GATA2 knockdown significantly impaired AR recruitment to these genomic sites, similarly to the effects observed with AR knockdown (Fig. 1J), confirming GATA2 in promoting AR chromatin reassociation and Daro resistance.

Conversely, overexpressing GATA2 in LNCaP cells led to increased cell growth and Daro resistance, suggesting GATA2's key role in resistance development (Fig. 1K; Fig. S6A). Similar effects were observed in 22Rv1 and VCaP cells (Fig. S6B–E). Treatment with K7174, a GATA2 inhibitor, sensitized LNCaP cells to Daro, suggesting GATA2 can potentially be a therapeutic target to overcome Daro resistance (Fig. 1L). Furthermore, DaroR cells showed resistance to other second-generation antiandrogens, such as enzalutamide and apalutamide, unlike parental LNCaP cells, indicating a broad loss of antiandrogen sensitivity (Fig. 1M and N).

Our study identifies an important Daro resistance mechanism: increased GATA2 levels facilitate AR chromatin reassociation, enhancing resistant cell growth. Figure 1O illustrates that in parental cells, AR chromatin binding is constrained by limited GATA2. As cancer cells evolve, they increase GATA2 expression, enabling efficient AR chromatin binding in resistant cells by increasing chromatin accessibility. This co-binding of GATA2 and AR potentially stabilizes the transcriptional activation complex and promotes gene expression. Collectively, this study demonstrates the critical role of GATA2 in Daro resistance and its potential as a promising therapeutic target.

## Funding

This work was supported by the 10.13039/100000002US National Institutes of Health (No. R33AI133697 to Q.F.; R01CA211861 to B.H.).

## Author contributions

Q.F. and B.H. conceptualized the project. Q.F. and T.Z. planned and designed the project. T.Z., C.Y., and Y.H. performed the experiments. Q.F. and T.Z. wrote the manuscript. All authors helped in the discussion and revision of the manuscript.

## Data availability

The sequencing data reported in this study has been deposited to the GEO database (GSE249437), with ChIP-sequencing data (GSE249435) and RNA-sequencing data (GSE249436). All other data are available in the main text or the supplementary materials.

## Conflict of interests

The authors declared no conflict of interests.
