# Supplementary material for: GATA2 up-regulation restores androgen receptor chromatin association and advances darolutamide resistance in prostate cancer
Source: Genes Dis. 2024 Dec 28;12(4):101508. doi: 10.1016/j.gendis.2024.101508 (PMC11978331; doi:10.1016/j.gendis.2024.101508)
Supplement: Multimedia component 1 [file mmc1.docx]

**Supplementary Data**

1. **Supplementary Figures**

**Figure S1.** AR Cistrome in darolutamide-resistant LNCaP Cells. ***(A)*** Phase contrast microscopy images of LNCaP parental and darolutamide-resistant (DaroR) cells. ***(B)*** Expression levels of the AR protein in darolutamide-resistant LNCaP cells. The parental LNCaP cells were subjected to a treatment of DMSO (Vehicle), 1nM R1881 (R1881), or 10 µM darolutamide (Daro) for 24h prior to the collection of the cells. ***(C)*** An Integrative Genomics Viewer (IGV) snapshot displaying AR ChIP-seq tracks for *FKBP5, KLK2,* and *ALCAM* genes. ***(D)*** Quantitative assessment of AR occupancy on the enhancer/promoter regions of the specified genes using ChIP-qPCR. N=3. The relevant target peaks are marked with grey arrows in (C). ***(E)*** Quantification of *FKBP5, KLK2*, and *ALCAM* mRNA levels by RT-qPCR analysis. N=3. All error bars are represented as SD.

**Figure S2.** Characterization of DaroR-Specific Genes and Pathways via RNA Sequencing Analysis. (***A***) A heatmap depicting the differential gene expression, including both upregulated and downregulated genes, in DaroR cells compared to the vehicle-treated parental cells by RNA-seq. The p-value threshold is set at 0.05. (***B***) A heatmap illustrating mRNA expression levels of androgen-responsive genes by RNA-seq. (***C***) Gene Set Enrichment Analysis (GSEA) reveals substantial restoration of androgen-responsive gene expression in DaroR cells. (***D***) Gene Ontology (GO) enrichment analysis of differentially expressed genes activated in DaroR cells based on RNA-seq results. (***E***) GO enrichment analysis of genes displaying DaroR-specific AR binding sites, as determined by ChIP-seq results. (***F***) IGV snapshot displaying AR ChIP-seq tracks for *SEMA3C* gene. (***G***) AR binding to *SEMA3C* gene determined by ChIP-qPCR analysis. The examined gene locus is marked by a grey arrow in (F). (***H***) Relative *SEMA3C* mRNA expression levels determined by RT-qPCR. N=3. The error bars are represented as SD.

**Figure S3**. *GATA2* expression in VCaP and 22Rv1 cells upon long-term (3 months) darolutamide treatment. (***A***) Relative *GATA2* mRNA levels in VCaP and 22Rv1 cells were determined by RT-qPCR. (***B***) GATA2 protein levels in VCaP and 22Rv1 cells were determined by Western blot analysis. The italicized numbers labeled at the bottom represent quantification via ImageLab (BioRad), with normalization to β-actin levels.

**Figure S4**. The expression of GATA2 in enzalutamide-resistant cells and prostate cancer tissues. ***(A)*** GATA2 mRNA expression in LNCaP cell models of enzalutamide resistance. The RNA-seq data were downloaded from the GEO archive under accession number GSE137833. EnzR: cells were made resistant to enzalutamide by long-term culture (> 6 months) in enzalutamide. V7, AR splicing variant V7-overexpressed cells. ***(B)*** GATA2 expression in benign (N=8), primary (N=15), and advanced prostate cancer and CRPC tissues (N=19). The RNA-seq data were downloaded from the GEO archive under accession number GSE80609.

**Figure S5**. Impact of GATA2 ablation on the expression of AR-regulated genes in darolutamide-resistant (DaroR) cells. ***(A)*** GATA2 or AR knockdown in DaroR cells by siRNA. Cells were collected 48h post-siRNA transfection, and the knockdown efficiency was evaluated by RT-qPCR analyses of GATA2 and AR mRNA levels. N=3, error bars: SD. ***(B)*** Western blot analysis of GATA2 and AR protein levels following siRNA treatment. Italicized numbers at the bottom represent the quantification of the signals by ImageLab (BioRad), normalized to β-actin levels. ***(C)*** KEGG pathways derived from the differentially downregulated genes in siGATA2 cells. ***(D)*** KEGG pathways derived from the differentially downregulated genes in siAR cells.

**Figure S6**. GATA2 upregulation contributes to antiandrogen resistance. ***(A-C)*** Generation of LNCaP, 22Rv1, and VCaP cells stably expressing Flag-tagged GATA2. The control (Ctrl) cells were created using the lentiviral backbone vector. Western blot demonstrates GATA2 protein expression. ***(D)*** Cell growth assay of GATA2-overexpressing 22Rv1 cells and their paired control cells, which were treated with either DMSO (vehicle) or 10μM darolutamide (Daro), with cell growth determined on the indicated days ***(E)*** Same assay as shown in (D) except that VCaP cells were used.

1. **Materials and methods**

***Cell culture***

LNCaP and 22Rv1 cells were cultured in RPMI 1640 medium supplemented with 10% FBS with 10% FBS and 1% penicillin/streptomycin. To produce the darolutamide-resistant LNCaP cells, LNCaP cells were exposed to gradually increasing concentrations of darolutamide (Targetmol, USA) until the concentration of 10 μM was attained. These DaroR cells were maintained in culture media with 10 μM darolutamide for 12 months to confirm the establishment of drug resistance. DaroR cells were cultured in other second-generation anti-androgen drugs enzalutamide and apalutamide (Selleckchem, USA). LNCaP cells drug sensitivity test was done with both darolutamide and GATA2 inhibitor K7174 (Sigma-Aldrich, USA). VCaP were cultured in DMEM with 10% FBS and 1% penicillin/streptomycin. All cells were grown at 37°C in a humidified atmosphere at 5% CO_2_. Mycoplasma contamination testing has been performed every six months. The cell images were taken using BioTek Cytation 5.

***siRNA and transient transfection***

ON-TARGETplus SMARTpool siRNA (Horizon) was used for siRNA knockdown using the Lipofectamine RNAiMAX Reagent (Thermo Fisher Scientific, # 13778075, USA) following the manufacturer’s instructions. Cells were transfected with GATA2 siRNA or AR siRNA with a final concentration of 20 nM. The efficiency of each siRNA was confirmed by RT-qPCR and Western blot analysis. All other transient transfection experiments were performed using PEI-Max (Polysciences, USA).

***Cell viability assay***

Prostate cancer cells were seeded at a density of 1 × 10^5^ cells per well in flat-bottomed 96-well plates. CellTiter-Glo® Luminescent Cell Viability Assay (Promega) was used to measure cell viability every other day following the manufacturer’s instructions. Cell viability was determined by measuring the luminescence using the Synergy™ neo2 multi-mode reader (BioTek).

***RT-qPCR assay***

To assess relative gene expression, we employed the RT-qPCR assay. Prostate cancer cells were exposed to different ligands for a duration of 24h. Subsequent to this treatment, total RNAs were extracted utilizing the RNeasy Plus Kit (Qiagen, #74034, USA) and quantified using the BioTek Synergy Neo2. 1µg of total RNA from each sample was used for reverse transcription by the iScript cDNA Synthesis Kit (BioRad). These cDNAs were used for qPCR using PerfeCTa qPCR Supermix (QuantaBio, #95113-012) on the Thermo ABI 7500 Fast Real-time qPCR system (Applied Biosystems, #4407205, USA).

***Western blot and antibodies***

Prostate cancer cells were collected and lysed with cold RIPA buffer (Sigma) containing 1mM PSMF(Sigma) and 1X Complete protease inhibitors (Takara Bio). For Western blot analysis, an equal amount of total protein was separated using precast polyacrylamide gradient gels (Bio-Rad). After protein transferring and blocking, the membranes were incubated with primary antibodies (1:1 000) at 4°C overnight and then with HRP-conjugated secondary antibodies at room temperature for 1h. Blots were developed by chemiluminescence (ECL) reagent, and the signals were visualized by the ChemiDoc™ MP equipped with Image Lab™ 6.0.1 Software (BioRad). The commercial antibodies used for Western blot analysis include: GATA2 (#sc9008H), AR (N20, #sc816), and β-Actin antibodies (#sc-47778) were obtained from Santa Cruz Biotechnology (USA), and FLAG M2 HRP antibody was from Sigma-Aldrich.

***RNA-seq analysis***

Total RNA was extracted using the RNeasy Plus Kit (Qiagen). RNA quality control (QC) was assessed using the Bioanalyzer (Agilent). RNA samples that met the quality criteria were sent to Novogene for both library preparation and sequencing via the Illumina NextSeq550 platform. Raw FASTQ files were generated and subsequently filtered using the Trim-galore tool. The filtered FASTQ files were aligned using STAR 2.7, referencing the human genome hg19 (GRCh37) and the gencode.v19 comprehensive gene annotation file[1]. FeatureCounts was used to count gene fragments based on the gencode.v19 comprehensive gene annotation file. Differential gene expression analysis was conducted using DESeq2, with the output from FeatureCounts as input[2]. Genes were considered significant if they had a p-value less than 0.05 and a base mean expression greater than 50. Data visualization, through volcano plots and heatmaps, was performed using ggplot2. Gene Set Enrichment Analysis (GSEA) was carried out using the GSEA desktop software, focusing on the ranking of genes within the enrichment of the MsigDB Collection and specific Androgen response pathways[3, 4]. The R package ClusterProfiler was used for enrichment pathway analysis[5]. Differentially expressed genes were analyzed using ClusterProfiler to evaluate their roles in biological processes, referencing both the GO_term and KEGG pathway databases.

***Gene expression and ChIP-seq analysis from existing GEO studies***

We utilized previously published data from the Gene Expression Omnibus (GEO) database for our study. Specifically, we downloaded gene expression count data from GEO study GSE137833[6]. EnzR: cells were made resistant to enzalutamide by long-term culture (> 6 months) in enzalutamide. V7, AR splicing variant V7-overexpressed cells. After normalizing for reading depth, we conducted correlation clustering analysis using DESeq2. Additionally, data from GEO study GSE80609 was downloaded, normalized and processed using DESeq2[7]. GATA2 expression was plotted before and after androgen deprivation therapy. We used GenomicRanges and ChIPPeakAnno for overlap analysis[8, 9], while matrix computations, heatmap creation, and coverage map processing were performed with deepTools.

**ChIP-sequencing and ChIP-qPCR assay**

The ChIP assay was carried out with the ChIP-IT High Sensitivity Kit (Active Motif, #53040, USA), following the manufacturer's instructions. LNCaP cells were treated with either a vehicle (DMSO), 10 nM R1881, or 5µM darolutamide for 4h, while DaroR cells were directly fixed. The AR-antibody (Active Motif) was utilized for all the ChIP experiments. ChIP-sequencing was performed by Novogene, generating the FASTQ files that were subsequently trimmed using Trim-galore. The processed data were then aligned to the hg19 (GRCh37) reference genome utilizing Bowtie2[10]. Bigwig files were created from the Bowtie2 output (Bam files) using deepTools, allowing for visualization in IGV[11]. Peak calling was performed using MACS2, following a standardized transcription factor ChIP-seq code[12]. Heatmaps and coverage maps were generated using deepTools. The output from MACS2 was analyzed with ChIPSeeker[13] and ChIPPeakAnno for overlapping and gene annotations[9]. The annotated genes were analyzed further using ClusterProfiler and DAVIDpathways[14]. For ChIP-qPCR, we used PerfeCTa qPCR supermix (QuantaBio, #95113-012). The sequencing data reported in this study has been deposited to the GEO database (access number GSE249437), with ChIP-seq data (GSE249435) and RNA-seq data (GSE249436).

***Statistical analysis***

Data in this study were analyzed using Prizm 8.0 (GraphPad, San Diego, CA, USA). The sample size was set to a minimum of three independent experiments (biological repeats) and experimental findings were reliably reproducible. Statistical significance of multiple samples was determined by one-way ANOVA, and between two groups was determined by non-paired Student-*t* test (*P<0.05, **P<0.01, ***P<0.001, ****P<0.0001). The N numbers of biological replicates were indicated in the figure legends. Differences were considered statistically significant at p <0.05.

**References**

1 Dobin A, Davis CA, Schlesinger F, Drenkow J, Zaleski C, Jha S *et al*. STAR: ultrafast universal RNA-seq aligner. *Bioinformatics* 2013; 29: 15-21.

2 Love MI, Huber W, Anders S. Moderated estimation of fold change and dispersion for RNA-seq data with DESeq2. *Genome Biol* 2014; 15: 550.

3 Liberzon A, Subramanian A, Pinchback R, Thorvaldsdottir H, Tamayo P, Mesirov JP. Molecular signatures database (MSigDB) 3.0. *Bioinformatics* 2011; 27: 1739-1740.

4 Nelson PS, Clegg N, Arnold H, Ferguson C, Bonham M, White J *et al*. The program of androgen-responsive genes in neoplastic prostate epithelium. *Proc Natl Acad Sci U S A* 2002; 99: 11890-11895.

5 Wu T, Hu E, Xu S, Chen M, Guo P, Dai Z *et al*. clusterProfiler 4.0: A universal enrichment tool for interpreting omics data. *Innovation (Camb)* 2021; 2: 100141.

6 Kregel S, Wang C, Han X, Xiao L, Fernandez-Salas E, Bawa P *et al*. Androgen receptor degraders overcome common resistance mechanisms developed during prostate cancer treatment. *Neoplasia* 2020; 22: 111-119.

7 Yun SJ, Kim SK, Kim J, Cha EJ, Kim JS, Kim SJ *et al*. Transcriptomic features of primary prostate cancer and their prognostic relevance to castration-resistant prostate cancer. *Oncotarget* 2017; 8: 114845-114855.

8 Lawrence M, Huber W, Pages H, Aboyoun P, Carlson M, Gentleman R *et al*. Software for computing and annotating genomic ranges. *PLoS Comput Biol* 2013; 9: e1003118.

9 Zhu LJ, Gazin C, Lawson ND, Pages H, Lin SM, Lapointe DS *et al*. ChIPpeakAnno: a Bioconductor package to annotate ChIP-seq and ChIP-chip data. *BMC Bioinformatics* 2010; 11: 237.

10 Langmead B, Salzberg SL. Fast gapped-read alignment with Bowtie 2. *Nat Methods* 2012; 9: 357-359.

11 Ramirez F, Ryan DP, Gruning B, Bhardwaj V, Kilpert F, Richter AS *et al*. deepTools2: a next generation web server for deep-sequencing data analysis. *Nucleic Acids Res* 2016; 44: W160-165.

12 Zhang Y, Liu T, Meyer CA, Eeckhoute J, Johnson DS, Bernstein BE *et al*. Model-based analysis of ChIP-Seq (MACS). *Genome Biol* 2008; 9: R137.

13 Wang Q, Li M, Wu T, Zhan L, Li L, Chen M *et al*. Exploring Epigenomic Datasets by ChIPseeker. *Curr Protoc* 2022; 2: e585.

14 Sherman BT, Hao M, Qiu J, Jiao X, Baseler MW, Lane HC *et al*. DAVID: a web server for functional enrichment analysis and functional annotation of gene lists (2021 update). *Nucleic Acids Res* 2022; 50: W216-W221.
